# Discussing sexuality with patients with Parkinson’s disease: a survey among Dutch neurologists

**DOI:** 10.1007/s00702-016-1655-x

**Published:** 2016-11-24

**Authors:** Petra J. M. van Hees, Anton A. van der Plas, Gaby F. van Ek, Hein Putter, Brenda L. Den Oudsten, Marjolein E. M. den Ouden, Henk W. Elzevier

**Affiliations:** 1 Department of Neurology, Alrijne Hospital, Simon Smitweg 1, 2353 GA Leiderdorp, The Netherlands; 2 Department of Urology, Leiden University Medical Center, Albinusdreef 2, PO Box 9600, 2300 RC Leiden, The Netherlands; 3 Department of Medical Statistics, Leiden University Medical Center, Albinusdreef 2, 2300 RC Leiden, The Netherlands; 4 Department of Medical and Clinical Psychology, Tilburg University, Warandelaan 2, 5037 AB Tilburg, The Netherlands; 5 Research Center of Nursing, Saxion University of Applied Sciences, M.H. Tromplaan 28, 7513 AB Enschede, The Netherlands

**Keywords:** Parkinson’s disease, Practice patterns, Sexual dysfunction, Questionnaire

## Abstract

Sexual functioning is often impaired in patients with Parkinson’s disease (PD) and may affect quality of life of patients and their spouse. However, little is known about the practice patterns of neurologists with regard to discussing sexuality in this field. The aim of this cross-sectional study was to evaluate to what extent neurologists discuss sexuality with PD patients. A 22-item questionnaire was sent to 139 neurologists specializing in PD. The survey contained questions about their attitudes, knowledge, and practice patterns with respect to sexual dysfunction (SD) in patients with PD. The response rate of the survey was 66.9%. Most participants (56.8%) stated that they address sexuality in less than half of their PD patients. High age of patients (42.0%), insufficient consultation time (37.5%), and a lack of patients’ initiative to raise the topic themselves (36.4%) were frequently reported barriers towards discussing sexuality. The majority of participants considered that discussing sexuality is a responsibility that lay with neurologists (85.2%), nurses (73.9%), and patients (72.7%). One quarter of the neurologists reported to have insufficient or no knowledge on SD. The majority of participants regarded screening for SD important or slightly important (85.2%). A large proportion of Dutch neurologists specializing in PD do not routinely discuss sexuality with their PD patients. Sexual healthcare in PD patients may benefit from time-efficient tools and agreements on who is responsible for discussing SD. Furthermore, recommendations in PD guidelines on screening and managing SD should be adapted to fit everyday practice.

**Electronic supplementary material:**

The online version of this article (doi:10.1007/s00702-016-1655-x) contains supplementary material, which is available to authorized users.

## Introduction

Parkinson’s disease (PD) is a heterogeneous neurodegenerative disorder characterized by a gradual appearance of motor and non-motor symptoms (NMS) (Jankovic 2008). Although the diagnosis of idiopathic PD is based on classical motor features of parkinsonism (National Collaboration Centre for Chronic Conditions UK 2006), PD patients frequently develop NMS prior to the onset of motor complaints (Breen and Drutyte 2013; Chaudhuri et al. 2006, 2011; Chaudhuri and Odin 2010). The spectrum of NMS is broad and includes sexual dysfunction (SD) (Chaudhuri et al. 2006, 2011; Chaudhuri and Odin 2010). SD in PD may either be an intrinsic feature of the disease or result indirectly from PD-related motor symptoms that interfere with intimate touch and sexual (Bronner 2011).

SD is a frequent problem among PD patients. A recent study demonstrated altered interest in sex in 57% of PD patients and problems during sexual intercourse in 66% of PD patients (Santos-García and de la Fuente-Fernández 2013). The presentation of SD is diverse. Difficulties reaching orgasms are more common in PD patients compared to matched controls (Bronner et al. 2004; Koller et al. 1990; Sakakibara et al. 2001). Erectile dysfunction and ejaculation problems are reported in 79% of male PD patients (Bronner et al. 2004; Koller et al. 1990; Sakakibara et al. 2001). In women with PD, involuntary urination during coitus and vaginal tightness are more prevalent compared to matched controls (Welsh et al. 1997). Furthermore, loss of lubrication is indicated as a problem in female PD patients (Koller et al. 1990; Kotková and Weiss 2013). PD patients may also experience SD as a side effect of dopaminergic treatment (Nakum and Cavanna 2016). SD induced by dopaminergic treatment often concerns hypersexuality or compulsive sexual behavior instead of hyposexual problems (Nakum and Cavanna 2016). Hypersexuality occurs in approximately 2.7% of PD patients who receive dopaminergic treatment and in 7.4% of PD patients who receive a dopamine agonist (Nakum and Cavanna 2016). Impaired sexual functioning in PD patients contributes to a reduced frequency of sexual intercourse or even sexual abstinence (23% in male and 22% in female patients) (Bronner et al. 2004; Sakakibara et al. 2001). This suggests that SD in PD patients also impedes the sexual health of their partners. In fact, partners of PD patients experience sexual dissatisfaction as much as patients do (Wielinski et al. 2010).

It is assumed that SD in general has a negative influence on quality of life (QoL) (Laumann et al. 1999). In PD, erectile dysfunction, impaired sex drive, and reduced libido have a significant impact on patients’ well-being (Baig et al. 2015; Duncan et al. 2014). Unfortunately, sexual functioning is barely assessed in disease-specific questionnaires, such as the PDQ39 (Chaudhuri et al. 2006).

Treatment strategies for SD in PD patients are limited. Sildenafil, a PDE-5 inhibitor, has proved to be effective in the treatment of erectile dysfunction (Zesiewicz et al. 2010). Dose reduction or discontinuation of a dopamine agonist is recommended when patients experience hypersexuality (Nakum and Cavanna 2016). Despite the lack of therapeutic options, discussing sexuality with PD patients remains essential, considering the broad diversity of SD, the high prevalence, and impact on the well-being of patients and their spouse. However, to our knowledge, no study has yet been published that has focused on neurologists’ practice patterns with respect to SD in their PD patients.

The aim of this study was to evaluate to what extent neurologists specializing in PD discuss sexuality with their PD patients. This study also focused on existing barriers towards discussing SD, neurologists’ perspective on who is responsible for discussing SD, their level of knowledge, and the need for additional training to extend their knowledge regarding SD. In addition, information was obtained about the use of the Parkinson Monitor (Parkinson’s Well-Being Map), a tool that allows PD patients to record both motor symptoms and NMS, including SD (see Online Resource 1). Furthermore, it was determined which variables were associated with the frequency of discussing sexuality.

## Materials and methods

### Study design and procedure

A cross-sectional study was performed among all Dutch neurologists specializing in PD. All neurologists who are registered at ParkinsonNet (*n* = 139) received a questionnaire in March 2016. ParkinsonNet is a national platform for PD healthcare professionals that offers educational programs, enhances collaboration, and facilitates referral to other PD care providers (ParkinsonNet). Questionnaires along with information letters were sent by regular post. The questionnaire could be returned anonymously in the enclosed (post-paid) retour envelope. Using numbered questionnaires, the response was monitored. A reminder to non-respondents was sent 4 weeks after the initial questionnaires were sent. Six weeks later, a final reminder was sent to neurologists who did not respond to the first reminder.

Following the Dutch guidelines of medical research, no formal ethical approval was needed.

### Survey design

The questionnaire was based on questionnaires used in previous studies concerning the discussion of sexuality in other medical departments (Korse et al. 2016; Krouwel et al. 2015; Nicolai et al. 2013; van Ek et al. 2015). With the help of an academic neurologist specializing in PD and the Dutch PD Patient Association, the questionnaire was adjusted to fit the population of our study. The questionnaire consisted of 22 questions (see Online Resource 2). Main topics in the questionnaire were the frequency and barriers of discussing sexuality. In addition, information was obtained about perspectives on responsibilities for discussing SD, level of knowledge on SD, the use of the Parkinson Monitor, possibilities for referral, and the need for training to extend knowledge on SD. The questionnaire also contained questions concerning demographic data. Respondents were offered an option to reject participation and were asked for reasons why they refused to participate.

### Statistical analyses

Quantitative data were analyzed using SPSS Statistics 23 (SPSS Inc., Chicago, IL, USA). Descriptive statistics were used to describe demographic variables and answers to questions. As the Shapiro–Wilks test showed a non-normal distribution of numerical data, the results were described as median [interquartile range (IQR)] and non-parametric analysis was applied. Mann–Whitney tests were performed to assess associations between numerical data of two groups. Associations between categorical data were calculated using Fisher’s exact tests. Adjustment for multiple testing was done by using the Bonferroni correction. Two-sided *p* values of <0.05 were considered statistically significant. Some answers were grouped together for analyses. The response options for questions 7, 8, 9, and 17 were clustered into a smaller number of outcome categories to make a clearer distinction between groups; for example, response options ‘In less than half of the cases’ and ‘Never/almost never’ were grouped together and defined ‘In less than half of the cases’.

## Results

### Survey responses

Of the 139 eligible respondents, 93 (66.9%) returned the survey. Three respondents were not willing to participate. Two of them returned the questionnaire without specifying a reason, and the third neurologist stated ‘lack of time’ as the reason. Two questionnaires were excluded from analyses, because they were completed by nurses. As such, 88 questionnaires were considered suitable for analysis.

### Demographics

Demographic data are presented in Table 1. Male participants (median 48, IQR 40–57) were significantly older than female participants (median 42, IQR 38–47; *p* = 0.004). Respondents (*n* = 93) and non-respondents (*n* = 46) did not differ with respect to the type of hospital they work in (*p* = 0.385). Gender and age of non-respondents were unknown.

**Table 1:** Demographic characteristics of participants (*n* = 88)

Gender, *n* (%)	
Male	56 (63.6)
Female	32 (36.4)
Age in years, median (IQR)^a^	44.5 (40.0-53.5)
Time of practice in neurology, *n* (%) (years)	
<1	0 (0)
1–2	9 (10.2)
3–5	13 (14.8)
6–10	27 (30.7)
11–15	13 (14.8)
>15	26 (29.5)
Clinical setting^b^, *n* (%)	
Tertiary or university hospital	12 (13.6)
General hospital	76 (86.4)
Specialized hospital	0 (0)
Unknown	2 (2.3)

^a^ *IQR* interquartile range

^b^Exceeds 100% because multiple answers were possible

### Discussion of sexuality

Neurologists were asked how often they discuss sexuality with PD patients without taking the age or gender of the patient into account (Table 2). The same question was asked for separate age groups and gender (Table 2). Nineteen neurologists (21.6%) reported that sexuality is addressed by the PD nurse or counselor. Sexuality is discussed less often with female PD patients (*p* < 0.0001) and with PD patients over the age of 70 years (Bonferroni adjusted *p* < 0.01 compared to other age groups). The extent to which sexuality is discussed was also evaluated for patient categories based on the use and efficacy of antiparkinsonian drugs and the presentation of NMS (Table 3).

**Table 2:** Discussing sexuality with PD patients, total results and results in subgroups according to gender and age

	In less than half of the cases^a^ *n* (%)	In half of the cases *n* (%)	In more than half of the cases^b^ *n* (%)	This is done by someone else *n* (%)
Total^c^	50 (56.8)	17 (19.3)	14 (15.9)	19 (21.6)
Male patients	49 (55.7)	20 (22.7)	19 (21.6)	NA^d^
Female patients	71 (80.7)	9 (10.2)	8 (9.1)	NA^d^

Years	Never	Seldom	Regularly	Often
30–40^e^	0 (0)	35 (44.9)	37 (47.4)	6 (7.7)
40–50^e^	0 (0)	36 (41.9)	43 (50.0)	7 (8.1)
50–60^e^	2 (2.3)	36 (41.4)	43 (49.4)	6 (6.9)
60–70^e^	10 (11.5)	39 (44.8)	33 (37.9)	5 (5.7)
>70^e^	14 (16.1)	48 (55.2)	21 (24.1)	4 (4.6)

^a^‘In less than half of the cases’ contains answers ‘Never/almost never’ and ‘In less than half of the cases’

^b^‘In more than half of the cases’ contains answers ‘In more than half of the cases’ and ‘Almost always/always’

^c^Exceeds 100% because multiple answers were possible

^d^Not applicable

^e^N differs, because some questions were skipped or forgotten

**Table 3:** Discussing sexuality with PD patients, in subgroups according to medication and presentation of NMS

Condition	*n* (%)^a^
Patients using a dopamine agonist	68 (77.3)
Patients with a lot of non-motor symptoms	40 (45.5)
Patients not using any antiparkinsonian drugs	26 (29.5)
Patients using antiparkinsonian drugs other than a dopamine agonist	25 (28.4)
Other^b^	22 (25.0)
Patients with poor motor response to medication	20 (22.7)
Patients with a good motor response to medication	19 (21.6)
Never	9 (10.2)

^a^Exceeds 100% because multiple answers were possible

^b^In case of ‘Other’, neurologists mentioned ‘If there is an angle or motive for asking (*n* = 11), ‘In all cases’ (*n* = 6), ‘In male patients’ (*n* = 2), ‘When patients initiates the subject’ (*n* = 2) and ‘Dependent on patient’s age’ (*n* = 1)

It was assessed whether the frequency of discussing sexuality was associated with participants’ gender or age. No differences were found between female and male neurologists (*p* = 0.3). Older participants (defined as age above median age) did not address sexuality differently compared to younger participants (defined as age below median age) (*p* = 0.141).

The majority of participants stated that they never or almost never (*n* = 42, 47.7%) or in less than half of the cases (*n* = 23, 26.1%) use the Parkinson Monitor during consultation. Nine neurologists (10.2%) responded that they use this tool with half of their patients. Eleven neurologists (12.5%) reported that they use it in more than half of their patients. Three participants (3.4%) indicated that they (almost) always use the Parkinson Monitor. The use of the Parkinson Monitor was associated with a higher frequency of discussing sexuality (*p* = 0.005).

The majority of participants stated that PD patients never or almost never (*n* = 42, 47.7%) or in less than half of the cases (*n* = 38, 43.2%) express SD spontaneously; seven neurologists answered ‘in half of the cases’ (*n* = 7, 8.0%) and one participant (1.1%) answered ‘in more than half of the cases’.

### Barriers

Neurologists were asked what barriers prevent them from addressing sexuality (Table 4). The barrier participants agreed on most was patients’ old age (*n* = 37, 42.0%), followed by insufficient time (*n* = 33, 37.5%) and the lack of initiative patients showed to express SD (*n* = 32, 36.4%).

**Table 4:** Barriers towards discussing sexuality; sorted from most agreed on to least agreed on

	Agree^a^ *n* (%)	Indecisive *n* (%)	Disagree^b^ *n* (%)
High age of the patient	37 (42.0)	23 (26.1)	28 (31.8)
Insufficient time	33 (37.5)	27 (30.7)	28 (31.8)
Patients do not express sexual problems spontaneously	32 (36.4)	19 (21.6)	37 (42.0)
Barriers based on language/culture/religion^c^	21 (24.1)	22 (25.3)	44 (50.6)
Insufficient training/knowledge^c^	16 (18.4)	44 (50.6)	27 (31.0)
Patient is too ill to discuss sexuality	16 (18.2)	15 (17.0)	57 (64.8)
I feel uncomfortable to talk about sexuality	13 (14.8)	30 (34.1)	45 (51.1)
Patient is not ready to discuss sexuality	9 (10.2)	30 (34.1)	49 (55.7)
Age difference between yourself and the patient	6 (6.8)	8 (9.1)	74 (84.1)
Someone else is accountable for discussing sexuality^c^	5 (5.7)	16 (18.4)	66 (75.9)
Patient is of the opposite sex	5 (5.7)	9 (10.2)	74 (84.1)

^a^‘Agree’ contains answers ‘Totally agree’ and ‘Agree’

^b^‘Disagree’ contains answers ‘Totally disagree’ and ‘Disagree’

^c^N differs, because some questions were skipped or forgotten

### Screening

The majority of participants regarded screening for SD important (*n* = 42, 47.7%) or slightly important (*n* = 33, 37.5%). Ten participants (11.4%) considered this very important and one participant (1.1%) regarded screening for SD unimportant. The remaining two participants (2.3%) were indecisive. Neurologists who considered screening for SD an important issue discussed SD more frequently (*p* = 0.003).

### Knowledge

Fifty-six neurologists (63.6%) stated to have some knowledge regarding SD. Twenty neurologists (22.7%) believed to have insufficient knowledge, and two participants (2.3%) reported a complete lack of knowledge. Nine participants (10.2%) assessed their knowledge as sufficient, and one participant (1.1%) indicated to possess a lot of knowledge. The level of knowledge was not associated with the frequency of discussing sexuality (*p* = 0.672). More than half of the participants (*n* = 50, 56.8%) confirmed that they need additional training to extend their knowledge to adequately discuss sexuality with PD patients.

### Responsibility

**Fig. 1:**
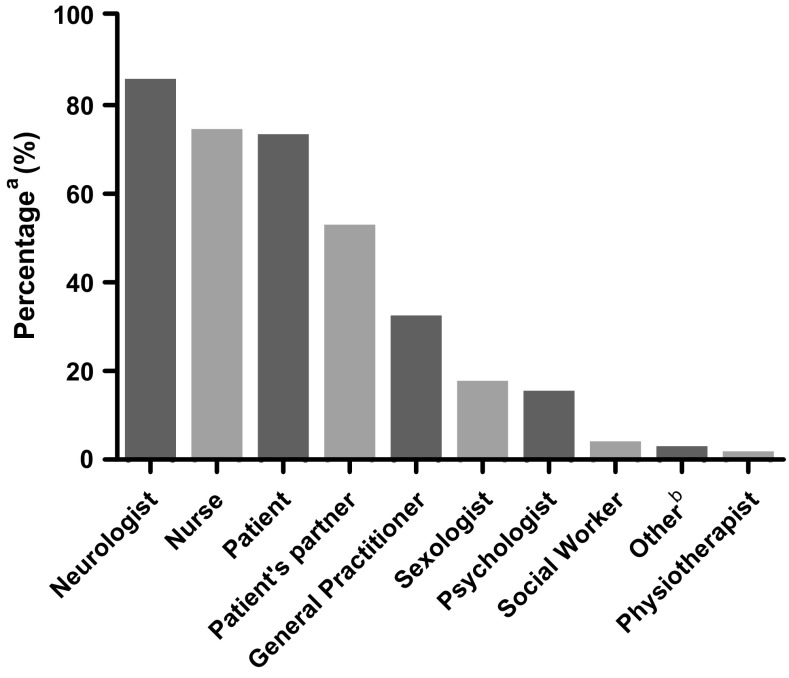
Responsibility for discussing sexuality. **a** Exceeds 100% because multiple answers were possible. **b** ‘Other’ includes ‘Collective responsibility’ (*n* = 1) and ‘Urologist’ (*n* = 1)

The majority of participants (*n* = 75, 85.2%) felt neurologists are responsible for addressing sexuality with patients. The nurse (*n* = 65, 73.9%), patient (*n* = 64, 72.7%), and the patient’s partner (*n* = 46, 52.3%) were also considered partly or fully responsible (Fig. 1). The survey also contained the statement that neurologists are responsible for discussing sexuality in PD patients. The majority of participants agreed (*n* = 58, 65.9%) or totally agreed (*n* = 14, 15.9%). A minority of participants disagreed (*n* = 5, 5.7%) or totally disagreed (*n* = 3, 3.4%). The remaining eight neurologists (9.1%) were indecisive on this statement.

### Referral

Neurologists estimated that they referred 5% (IQR 1–10) of their patients to another care provider for sexual counseling in the past year. Sixty-eight neurologists (77.3%) stated it to be possible to refer patients with SD to specialized care providers within their own center: the sexologist (*n* = 14) and the urologist (*n* = 12) were mentioned most. Two neurologists (2.3%) were unaware of the possibility to refer patients with SD within their center. The remaining 18 neurologists (20.5%) reported that internal referral is not possible; four of them stated that they refer patients to a university hospital. Most participants (*n* = 72, 82.8%) thought it would be useful to have a list of care providers to whom patients with SD could be referred to.

## Discussion

The spectrum of PD-related NMS consists of many symptoms, including SD that may impair QoL (Duncan et al. 2014). As such, neurologists face the challenge to screen PD patients thoroughly for both motor symptoms and NMS. We asked Dutch neurologists specializing in PD about their practice patterns with regard to discussing sexuality with their PD patients. The majority of participants reported that they often omit discussing sexuality, especially with women and patients over the age of 70 years. To our knowledge, this is the first study that has examined the level of attention neurologists pay to the sexual well-being of PD patients. Interestingly, our results show a remarkable similarity with the outcome of an earlier study among patients with multiple sclerosis that also demonstrated an undervaluation of sexuality by their treating physicians (Hulter and Lundberg 1995). Moreover, the undervaluation of SD seems not to be confined to neurological care, as studies that focused on other medical disciplines also revealed a lack of routine screening for SD (Korse et al. 2016; Krouwel et al. 2015; Nicolai et al. 2013; van Ek et al. 2015).

Participants were asked which barriers they encounter when discussing sexuality. High age of patients was the most reported barrier. This is concordant with the results of studies in which neurosurgeons and surgical oncologists were asked about practice patterns with regard to sexual health of their patients (Korse et al. 2016; Krouwel et al. 2015). A possible explanation for this lack of attention for SD in the elderly may be that medical specialists assume that the majority of elderly patients are not sexually active and do not experience SD. Nicolosi et al., however, demonstrated that 21% of women and 53% of men between 70 and 80 years had sexual intercourse within the year prior to study entry (Nicolosi et al. 2004). Moreover, evidence suggests that ageing is an important risk factor for developing SD (Camacho and Reyes-Ortiz 2005). The undervaluation of SD in elderly patients, despite the high risk of SD in this group, may indicate that a large proportion of medical specialists is unaware of the important role sexuality plays in the life of elderly people. Broadening this perspective to a general level of knowledge about SD, the majority of the participating neurologists in our study stated that their expertise on SD is insufficient and confirmed that they need an additional training to extend their knowledge. Interestingly though, lack of knowledge was not considered an important barrier to discuss sexuality by most of the participants and the level of knowledge was not associated with the frequency in which sexuality is addressed during consultation visits. Similar results were found in a study that examined the discussion of sexuality in neurosurgical practices (Korse et al. 2016). Nonetheless, we recommend that neurologists optimize their knowledge on PD-related SD to provide the best care for their patients. As such, we advocate the implementation of an education course on SD in PD in the training programs of neurology residents. The inclusion of a chapter on SD in the Dutch PD guidelines, an initiative of the Dutch Neurology Society, is commendable (Bloem et al. 2010). However, we question whether the recommendations presented within this chapter are applicable to everyday practice. Unfortunately, our questionnaire did not contain questions on how well the neurologists maintain the guidelines.

A substantial proportion of participants reported insufficient time as a barrier for discussing sexuality. This was also a restricting factor for neurosurgeons, nephrologists, and surgical oncologists towards addressing SD with patients (Korse et al. 2016; Krouwel et al. 2015; van Ek et al. 2015). Nonetheless, the vast majority of neurologists considered themselves mainly responsible for discussing sexuality. As the broad spectrum of PD symptoms will not change and consultation time is limited, a time-efficient and pragmatic tool to assess the complete spectrum of PD symptoms may be useful. The Parkinson Monitor is an example of such a tool and allows patients to record both motor symptoms and NMS before they visit their neurologist. As a result, neurologists have the opportunity to focus on items that the patient indicates as most bothersome. In our study, neurologists who use the Parkinson Monitor were more likely to discuss sexuality. To avoid the time constraint that neurologists encounter, other caregivers, who are specialized in PD, especially the PD nurse, may be of assistance to enquire about SD in PD patients, and support them in managing their SD. Many participants felt PD nurses also responsible for discussing sexuality with PD patients. In contrast, only one-fifth of the participants stated that the discussion of sexuality is actually done by the nurse. These findings indicate a lack of clear agreements on responsibilities within neurological practices and emphasize the efforts that need to be made to improve this.

Many neurologists reported that the lack of patients’ initiative is another barrier for discussing sexuality and that patients are responsible for discussing SD as well. However, 90% of the participants stated that patients seldom report SD spontaneously. A previous study also showed that PD patients were unlikely to express sexual problems (Hand et al. 2010). The hesitation of both patients and neurologists to discuss sexuality is what may cause an ongoing circle of avoidance. Routine screening for SD, either by neurologists or other care providers, may break this vicious circle. In our study, SD was discussed more frequently when screening was considered an important issue, suggesting that the quality of the discussion on sexuality may improve by raising awareness on the importance of screening.

To improve the quality of sexual healthcare for PD patients, we need a clear picture of the extent of the problem of PD-related SD and the perspectives of other neurological care providers. These items need to be addressed in future studies. In addition, as the undervaluation of SD affects PD patients and their partners, new studies should also focus on their need for sexual counseling and how this could be implemented in current healthcare systems.

This study has a couple of limitations. First, the response rate of 66.9% may have caused non-response bias. A comparison between age and gender of respondents and non-respondents was not possible, because gender and age of non-respondents were unknown. To attain a higher response rate, reminders were sent to non-respondents after the initial sending. Nonetheless, the response rate of this study was above average compared to mean response rates in physician surveys (54%) (Asch et al. 1997). Second, bias may have occurred due to the self-reported character of the questionnaire, leading to a possible under- or overestimation of our results. To reduce this bias, anonymous questionnaires were provided. Third, a non-validated questionnaire was used. A validated questionnaire on the topic of PD-related SD does not exist as far as we know. However, the questionnaire was based on surveys used in similar studies amongst other healthcare providers (Korse et al. 2016; Krouwel et al. 2015; Nicolai et al. 2013; van Ek et al. 2015).

## Conclusion

The majority of Dutch neurologists specializing in PD do not discuss sexuality routinely with their PD patients. Reasons for this undervaluation are ambiguous, although patients’ advanced age and insufficient time during consultation are reported as important factors. Assessment tools, such as Parkinson Monitor, may overcome the barrier of time constraint. Sexual healthcare will likely benefit from clearer agreements on responsibilities between neurologists and other care providers. Although the impact of knowledge of SD on discussing sexuality in PD care remains unclear, we advocate the implementation of this topic in the neurology residency training. The inclusion of a chapter on SD in the current Dutch PD guidelines is praiseworthy, but in our opinion needs to be adapted to fit everyday practice. To enhance sexual healthcare in PD, we welcome studies that focus on perspectives of other PD care providers and PD patients.

## Electronic supplementary material

Below is the link to the electronic supplementary material.

**Online Resource 1** Additional information about the Parkinson Monitor (PDF 517 kb)

**Online Resource 2** Questionnaire (translated from Dutch) (PDF 91 kb)

## References

[CR1] Asch DA, Jedrziewski MK, Christakis NA (1997). Response rates to mail surveys published in medical journals. J Clin Epidemiol.

[CR2] Baig F, Lawton M, Rolinski M, Ruffman C, Nithi K, Evetts SG, Fernandes HR, Ben-Shlomo Y, Hu MT (2015). Delineating nonmotor symptoms in early Parkinson’s disease and first-degree relatives. Mov Disord.

[CR3] Bloem BR, Van Laar T, Keus SHJ, De Beer H, Poot E, Buskens E, Aarden W, Munneke M (2010). Multidisciplinaire richtlijn Ziekte van Parkinson.

[CR4] Breen KC, Drutyte G (2013). Non-motor symptoms of Parkinson’s disease: the patient’s perspective. J Neural Trans (Vienna).

[CR5] Bronner G (2011). Sexual problems in Parkinson’s disease: the multidimensional nature of the problem and of the intervention. J Neurol Sci.

[CR6] Bronner G, Royter V, Korczyn AD, Giladi N (2004). Sexual dysfunction in Parkinson’s disease. J Sex Marital Ther.

[CR7] Camacho ME, Reyes-Ortiz CA (2005). Sexual dysfunction in the elderly: age or disease?. Int J Impot Res.

[CR8] Chaudhuri KR, Odin P (2010). The challenge of non-motor symptoms in Parkinson’s disease. Prog Brain Res.

[CR9] Chaudhuri KR, Healy DG, Schapira AH (2006). Non-motor symptoms of Parkinson’s disease: diagnosis and management. Lancet Neurol.

[CR10] Chaudhuri KR, Odin P, Antonini A, Martinez-Martin P (2011). Parkinson’s disease: the non-motor issues. Parkinsonism Relat Disord.

[CR11] Duncan GW, Khoo TK, Yarnall AJ, O’Brien JT, Coleman SY, Brooks DJ, Barker RA, Burn DJ (2014). Health-related quality of life in early Parkinson’s disease: the impact of nonmotor symptoms. Mov Disord.

[CR12] Hand A, Gray WK, Chandler BJ, Walker RW (2010). Sexual and relationship dysfunction in people with Parkinson’s disease. Parkinsonism Relat Disord.

[CR13] Hulter BM, Lundberg PO (1995). Sexual function in women with advanced multiple sclerosis. J Neurol Neurosurg Psychiatry.

[CR14] Jankovic J (2008). Parkinson’s disease: clinical features and diagnosis. J Neurol Neurosurg Psychiatry.

[CR15] Koller WC, Vetere-Overfield B, Williamson A, Busenbark K, Nash J, Parrish D (1990). Sexual dysfunction in Parkinson’s disease. Clin Neuropharmacol.

[CR16] Korse NS, Nicolai MP, Both S, Vleggeert-Lankamp CL, Elzevier HW (2016). Discussing sexual health in spinal care. Eur Spine J.

[CR17] Kotková P, Weiss P (2013). Psychiatric factors related to sexual functioning in patients with Parkinson’s disease. Clin Neurol Neurosurg.

[CR18] Krouwel EM, Hagen JH, Nicolai MP, Vahrmeijer AL, Putter H, Pelger RC, Elzevier HW (2015). Management of sexual side effects in the surgical oncology practice: a nationwide survey of Dutch surgical oncologists. Eur J Surg Oncol.

[CR19] Laumann EO, Paik A, Rosen RC (1999). Sexual dysfunction in the United States: prevalence and predictors. JAMA.

[CR20] Nakum S, Cavanna AE (2016). The prevalence and clinical characteristics of hypersexuality in patients with Parkinson’s disease following dopaminergic therapy: a systematic literature review. Parkinsonism Relat Disord.

[CR21] National Collaboration Centre for Chronic Conditions UK (2006) Diagnosing Parkinson’s disease. In: Parkinson’s Disease: National Clinical Guideline for Diagnosis and Management in Primary and Secondary Care., vol NICE Clinical Guidelines, No. 35. Royal College of Physicians, London 21089238

[CR22] Nicolai MP, Both S, Liem SS, Pelger RC, Putter H, Schalij MJ, Elzevier HW (2013). Discussing sexual function in the cardiology practice. Clin Res Cardiol.

[CR23] Nicolosi A, Laumann EO, Glasser DB, Moreira ED, Paik A, Gingell C (2004). Sexual behavior and sexual dysfunctions after age 40: the global study of sexual attitudes and behaviors. Urology.

[CR24] ParkinsonNet. Optimale zorg voor Parkinson, dat is ons doel! http://www.parkinsonnet.nl/. Accessed 3 Jun 2016

[CR25] Sakakibara R, Shinotoh H, Uchiyama T, Sakuma M, Kashiwado M, Yoshiyama M, Hattori T (2001). Questionnaire-based assessment of pelvic organ dysfunction in Parkinson’s disease. Auton Neurosci.

[CR26] Santos-García D, de la Fuente-Fernández R (2013). Impact of non-motor symptoms on health-related and perceived quality of life in Parkinson’s disease. J Neurol Sci.

[CR27] van Ek GF, Krouwel EM, Nicolai MP, Bouwsma H, Ringers J, Putter H, Pelger RC, Elzevier HW (2015). Discussing sexual dysfunction with chronic kidney disease patients: practice patterns in the office of the nephrologist. J Sex Med.

[CR28] Welsh M, Hung L, Waters CH (1997). Sexuality in women with Parkinson’s disease. Mov Disord.

[CR29] Wielinski CL, Varpness SC, Erickson-Davis C, Paraschos AJ, Parashos SA (2010). Sexual and relationship satisfaction among persons with young-onset Parkinson’s disease. J Sex Med.

[CR30] Zesiewicz TA, Sullivan KL, Arnulf I, Chaudhuri KR, Morgan JC, Gronseth GS, Miyasaki J, Iverson DJ, Weiner WJ (2010). Practice parameter: treatment of nonmotor symptoms of Parkinson disease: report of the Quality Standards Subcommittee of the American Academy of Neurology. Neurology.

