# Supplementary material for: Discussing sexuality with patients with Parkinson’s disease: a survey among Dutch neurologists
Source: J Neural Transm (Vienna). 2016 Nov 24;124(3):361–8. doi: 10.1007/s00702-016-1655-x (PMC5310558; doi:10.1007/s00702-016-1655-x)
Supplement: Supplementary file 1 — Online Resource 1 Additional information about the Parkinson Monitor (PDF 517 kb) [file 702_2016_1655_MOESM1_ESM.pdf]

Parkinson Monitor (Parkinson's Well-Being Map)

The Parkinson Monitor (internationally referred to as Parkinson's Well-Being Map) is a tool that allows patients to record Parkinson's disease symptoms. It is not a validated instrument. It encompasses all aspects of Parkinson's disease, i.e. both motor and non-motor symptoms, and offers patients the opportunity to indicate to what extent they suffer from those symptoms and highlight which symptoms are of most concern. The tool allows patients to monitor their own conditions and is useful to prepare consultations with the patients' neurologists. This will help neurologists to understand how living with Parkinson's disease affects the patient. Furthermore, it enables neurologists and other Parkinson's disease care providers to focus on symptoms that the patient indicates as most troublesome.

The Parkinson Monitor can be completed either by hand (by downloading and printing a PDF file) or by fulfilling an online version that can be saved to a personal computer (see Figure).

Parkinson Monitor is available in several languages and countries.

References

ParkinsonVereniging, UCB (2013) Parkinson Monitor. https://www.parkinson-vereniging.nl/wat-doen-wij/persoonlijk-parkinson-dossier/parkinson-monitor. Accessed 13 May 2016

USB S.A. Belgium (2013) Parkinson's Well-Being Map^{tm}. http://www.ucb.com/patients/support-tools/well-being-map. Accessed 6 November 2016.

Figure. Parkinson Monitor

Parkinson Monitor™

Markeer het cijfer in het diagram dat het beste aangeeft hoeveel last u van uw klachten heeft.
U geeft een cijfer aan de categorie in zijn geheel (bijv. slaapstoornissen), niet aan de afzonderlijke klachten.

0 Geen last · 1 Weinig last · 2 Last · 3 Veel last · 4 Zeer veel last

DATUM: (DD/MM/JJ) ....

Stemming

- ☐ Ik heb steeds minder interesse in dingen
- ☐ Ik heb minder/geen plezier meer in de dingen die ik voorheen leuk vond
- ☐ Ik voel me ongelukkig
- ☐ Ik ben nerveus, angstig of raak in paniek
- ☐ Ik ben depressief
- ☐ Ik pieker veel
- ☐ Ik heb problemen in de relationele sfeer/met gezinsleden
- ☐ Andere: ....

Slaapstoornissen

- ☐ Ik heb problemen om 's avonds in slaap te vallen
- ☐ Ik heb problemen met doorslapen
- ☐ Ik heb problemen om weer in slaap te vallen zodra ik wakker ben geworden
- ☐ Ik ben 's ochtends moe
- ☐ Ik ben gedurende de dag vermoeid
- ☐ Ik dut regelmatig in op ongewenste momenten
- ☐ Andere: ....

Aandacht/Geheugen

- ☐ Ik kan mij niet concentreren gedurende activiteiten
- ☐ Ik spreek traag
- ☐ Ik ben vergeetachtig
- ☐ Ik heb problemen met het herinneren van namen, getallen, gebeurtenissen
- ☐ Ik heb moeite om op woorden te komen
- ☐ Ik heb moeite met initiatief nemen
- ☐ Andere: ....

Spijsvertering

- ☐ Ik heb moeite met slikken
- ☐ Ik heb last van overvloedig speeksel
- ☐ Ik moet vaak overgeven of voel mij ziek (misselijk)
- ☐ Ik heb last van constipatie (verstopping)
- ☐ Ik heb last van diarree
- ☐ Ik heb last van mijn maag
- ☐ Andere: ....

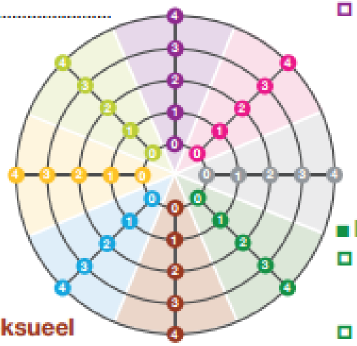

Andere niet-motorische symptomen

- ☐ Ik heb een licht gevoel in mijn hoofd/ben duizelig als ik opsta vanuit een liggende positie
- ☐ Ik val als gevolg van een flauwte
- ☐ Ik merk een wijziging in mijn vermogen om te ruiken/proeven
- ☐ Ik merk een wijziging in gewicht (niet te wijten aan een wijziging in dieet)
- ☐ Ik zweet overmatig
- ☐ Ik zie/hoor dingen die er niet zijn
- ☐ Andere: ....

Blaas en seksueel functioneren

- ☐ Ik voel aandrang om te plassen terwijl ik niet hoef te plassen
- ☐ Ik moet 's nachts vaker plassen
- ☐ Ik heb last van incontinentieproblemen
- ☐ Ik heb een gewijzigde interesse in seks
- ☐ Ik heb problemen bij het hebben van seks
- ☐ Andere: ....

Pijn

- ☐ Ik heb vroeg in de ochtend pijnlijke krampen in mijn tenen, vingers, enkels en polsen, waar ik wakker van word
- ☐ Ik heb pijnlijke, stijve ledematen gedurende de dag
- ☐ Ik heb pijnlijke, stijve ledematen gedurende de nacht
- ☐ Ik heb pijnschokken, -scheuten in mijn ledematen
- ☐ Ik heb pijn door abnormale ongewenste bewegingen
- ☐ Ik heb pijn door rusteloosheid of nerveus bewegen 's nachts
- ☐ Ik heb zware hoofdpijn
- ☐ Andere: ....

Beweging

- ☐ Ik heb last van stijfheid in de vroege ochtend
- ☐ Ik heb last van stijfheid
- ☐ Ik tril
- ☐ Ik beweeg traag
- ☐ Ik ben beperkt in mijn bewegingen
- ☐ Ik heb evenwichtsproblemen/valneigingen
- ☐ Ik heb een gewijzigde lichaamshouding
- ☐ Ik heb problemen met spreken
- ☐ Ik heb een klein handschrift
- ☐ Ik heb last van bevriezen (freezing)
- ☐ Andere: ....

Dit diagram en de gesommeerde klachten zijn niet gevalideerd.

Parkinson Monitor™

Het gesprek met mijn arts/verpleegkundige.

Door het invullen van de Monitor kunt u uw dagelijks functioneren vastleggen. Samen met uw behandelteam kunt u volgen hoe het met u gaat.
De Parkinson Monitor is bedoeld om het gesprek tussen u en uw behandelteam te vereenvoudigen.

De drie belangrijkste vragen die ik wil stellen aan mijn arts en/of verpleegkundige zijn:

1. ....
....
....
....

2. ....
....
....
....

3. ....
....
....

Zijn er tussen het vorige en het aankomende gesprek met de neuroloog nog veranderingen aangebracht in uw medicijngebruik (ander medicijn/dosering)? Indien ja, graag toelichting:

....
....
....
....
....
....
....
....
....
....
....

Receptvrije geneesmiddelen (bijv. Aspirine®)

....

Na afloop van het gesprek met mijn arts en/of verpleegkundige

Heb ik voldoende tijd gehad om al mijn vragen te stellen? ☐ Ja ☐ Nee

[illegible]

© UCB 2013. All rights reserved. Alle rechten zijn voorbehouden aan de Parkinson Vereniging en UCB. De Parkinson Monitor wordt gesponsord door UCB Pharma B.V.
