# Supplementary material for: Discussing sexuality with patients with Parkinson’s disease: a survey among Dutch neurologists
Source: J Neural Transm (Vienna). 2016 Nov 24;124(3):361–8. doi: 10.1007/s00702-016-1655-x (PMC5310558; doi:10.1007/s00702-016-1655-x)
Supplement: Supplementary file 2 — Online Resource 2 Questionnaire (translated from Dutch) (PDF 91 kb) [file 702_2016_1655_MOESM2_ESM.pdf]

**Questionnaire** (translated from Dutch)

*Reasons not to participate:*
- ☐ No time
- ☐ Not interested
- ☐ Not enough experience
- ☐ Research is not important in this area
- ☐ Other: ………………………………………

Please read the questionnaire carefully. Choose the most suitable answer at each question. Please do not skip any questions. In case of mistakes, please cross out the wrong answer and select the right box. Thank you in advance for your effort.

*Demographics*

1. What is your gender?
- o Male
- o Female

2. What is your age? _________ Years

3. Type of clinic/practice?
- o Tertiary or university hospital
- o General hospital
- o Specialized hospital (for instance a cancer center)

4. Time of practice in neurology?
- o < 1 year
- o 1-2 years
- o 3-5 years
- o 6-10 years
- o 11-15 years
- o > 15 years

*The following questions concern sexuality in Parkinson's disease*

5. In which patients suffering from Parkinson's disease do you discuss sexuality? (Multiple answers possible)
- ☐ Patients not using any antiparkinsonian drugs
- ☐ Patients using a dopamine agonist
- ☐ Patients using antiparkinsonian drugs other than a dopamine agonist
- ☐ Patients with good motor response to medication
- ☐ Patients with poor motor response to medication
- ☐ Patients with a lot of non-motor symptoms
- ☐ Never
- ☐ Other: ____________________________________________________________

6. In what percentage of patients suffering from Parkinson's disease do you think sexual function has changed?
_____________%

7. How often do you discuss sexual health with patients suffering from Parkinson's disease?
- o Never/almost never
- o In less than half of the cases
- o In half of the cases
- o In more than half of the cases
- o Almost always/always
- o This is done by someone else, namely: _______________________________

8. How often do you discuss sexual health with female patients suffering from Parkinson's disease?
- o Never/almost never
- o In less than half of the cases
- o In half of the cases
- o In more than half of the cases
- o Almost always/always

9. How often do you discuss sexual health with male patients suffering from Parkinson’s disease?
o Never/almost never
o In less than half of the cases
o In half of the cases
o In more than half of the cases
o Almost always/always

10. To what extent do you use the ‘Parkinson Monitor’ to assess non-motor manifestations, including sexual dysfunctions, in patients suffering from Parkinson’s disease?
o Never/almost never
o In less than half of the cases
o In half of the cases
o In more than half of the cases
o Almost always/always

11. What percentage of your patients suffering from Parkinson’s disease did you refer to another care provider for counselling of their sexual problems over the last year? _____________%

12. How often do patients express sexual problems spontaneously?
o Never/almost never
o In less than half of the cases
o In half of the cases
o In more than half of the cases
o Almost always/always

13. How often do you discuss sexual health with patients suffering from Parkinson’s disease in the age groups listed below?

| Age groups | Never | Seldom | Regularly | Often |
|---|---|---|---|---|
| 30-40 years | □ | □ | □ | □ |
| 40-50 years | □ | □ | □ | □ |
| 50-60 years | □ | □ | □ | □ |
| 60-70 years | □ | □ | □ | □ |
| > 70 years | □ | □ | □ | □ |

14. Who is, according to you, responsible for discussing sexuality? (Multiple answers possible)
□ Patient
□ Partner of the patient
□ Neurologist
□ General practitioner
□ Social worker
□ Nurse
□ Psychologist
□ Physiotherapist
□ Sexologist
□ Other, namely: ___________________________________________

15. How do you rate your own knowledge on sexual dysfunctions and the treatment of it?
o No knowledge at all
o Insufficient knowledge
o Some knowledge
o Sufficient knowledge
o A lot of knowledge

16. How often do you ask about the use of PDE5 inhibitors (Viagra, Levitra, Cialis) in male patients suffering from Parkinson’s disease?
o Never/almost never
o In less than half of the cases
o In half of the cases
o In more than half of the cases

o Almost always/always

17. Possible barriers towards discussing sexuality are listed below. To what extent are the barriers applicable to you? Please select only one answer for each barrier.

| | Totally disagree | Disagree | Slightly disagree/ slightly agree | Agree | Totally agree |
|---|---|---|---|---|---|
| I feel uncomfortable to talk about sexuality | □ | □ | □ | □ | □ |
| Insufficient time | □ | □ | □ | □ | □ |
| Insufficient training/knowledge | □ | □ | □ | □ | □ |
| Someone else is accountable for discussing sexuality | □ | □ | □ | □ | □ |
| Patient is not ready to discuss sexuality | □ | □ | □ | □ | □ |
| Patient is too ill to discuss sexuality | □ | □ | □ | □ | □ |
| Patients do not express sexual problems spontaneously | □ | □ | □ | □ | □ |
| Barriers based on language/culture/religion | □ | □ | □ | □ | □ |
| Patient is of the opposite sex | □ | □ | □ | □ | □ |
| High age of the patients | □ | □ | □ | □ | □ |
| Age difference between yourself and the patient | □ | □ | □ | □ | □ |

18. Is it possible to refer patients experiencing sexual problems to other care providers in your clinic?
o Yes
o No
o Unknown
If yes, to:__________________________________________________

*The following questions concern your opinion about discussing sexuality in Parkinson's disease*

19. The neurologist is responsible for discussing sexuality in Parkinson's disease.
o Totally disagree
o Disagree
o Indecisive
o Agree
o Totally agree

20. How important is it to screen for sexual dysfunction in Parkinson's disease?
o Unimportant
o Slightly important
o Important
o Very important
o Indecisive

21. Are you in need of extending your knowledge on discussing sexuality?
o Yes
o No

22. Are you in need of a directory of care providers to whom patients with sexual problems can be referred to?
o Yes
o No

---------------------------------------------------------------------------------------------------------------------------

*Thank you very much for completing the questionnaire.*
